# Perceived community environment and physical activity involvement in a northern-rural Aboriginal community

**DOI:** 10.1186/1479-5868-4-63

**Published:** 2007-12-04

**Authors:** Allison M Kirby, Lucie Lévesque, Virgina Wabano, Jennifer Robertson-Wilson

**Affiliations:** 1 Primary Healthcare Research Unit, Memorial University of Newfoundland, St. John's, Newfoundland, Canada; 2 School of Kinesiology and Health Studies, Queen's University, Kingston, Ontario, Canada; 3 Kahnawake Schools Diabetes Prevention Project, Kahnawake, Quebec, Canada; 4 A northern-rural Aboriginal Community, Ontario, Canada; 5 Department of Kinesiology and Physical Education, Wilfrid Laurier University, Kington, Ontario, Canada

## Abstract

**Background:**

Type 2 diabetes disproportionately affects Aboriginal peoples in Canada. Ample evidence shows that regular physical activity (PA) plays an important role in the prevention and treatment of type 2 diabetes. Evidence is beginning to emerge linking PA to the physical environment but little is known about the relationship between remote rural environments and PA involvement in Aboriginal peoples. This study's purpose was to investigate the relationship between perceptions of the environment and PA and walking patterns in Aboriginal adults in order to inform the planning and implementation of community-relevant PA interventions.

**Methods:**

Two hundred and sixty three residents (133 women, mean age = 35.6 years, SD = 12.3 and 130 men, mean age = 37.2 years, SD = 13.1) from Moose Factory, Ontario were asked about environmental factors related to walking and PA involvement. Survey items were drawn from standardized, validated questionnaires. Descriptive statistics (means, standard deviations, percentages) were calculated. A series of hierarchical multiple regressions were performed to determine associations between walking and overall PA with perceived environmental variables.

**Results:**

Hierarchical multiple regression to predict walking revealed significant associations between walking and perceived safety and aesthetics. Owning home exercise equipment predicted strenuous PA. Different aspects of the physical environment appear to influence different types of physical activities. The significant amount of variance in behaviour accounted for by perceived environmental variables (5.3% walking) included safety, aesthetics, convenience, owning home exercise equipment and comfortable shoes for walking.

**Conclusion:**

Results suggest that a supportive physical environment is important for PA involvement and that walking and activities of different intensity appear to be mediated by different perceived environmental variables. Implications for PA promotion in rural environments where Aboriginal people face many unique environmental features (e.g., bears, mosquitoes, extreme cold) are discussed.

## Background

The well-established connection between diabetes prevention and physical activity involvement [1-3] provides great impetus for promoting regular, physical activity involvement to sedentary or irregularly active persons at risk for diabetes. Initiatives to promote physical activity involvement increasingly encourage persons to become involved in moderate-intensity activities such as brisk-walking to achieve health benefits [4,5]. Walking is reported to be the most popular type of physical activity in the general population and also in major subgroups such as overweight persons, seniors, persons living on low incomes and persons with limited education [6-8]. Walking is noted for its acceptability, accessibility, low cost, low intensity, and its ease of performance particularly when compared with many other physical activities [9]. For this reason, participating in walking may alleviate many of the barriers individuals report when attempting to participate in other physical activities [10]. This study examines both walking and general physical activity given that walking may not always be perceived as exercise or physical activity [11] and physical activity may not always include walking.

The ability to alter one's activity level in general and walking in particular is dependent upon a multitude of correlates that mediate or directly influence physical activity involvement [12-16]. Identifying relevant correlates is essential for the development of effective interventions to increase population levels of walking and physical activity involvement [17,18]. Recent attention to potential environmental influences upon walking and physical activity involvement underlies the purpose of the current study to explore associations between walking and physical activity and perceptions of the environment. In particular, the recent push to identify correlates as they apply to persons who are at increased risk for physical inactivity (e.g., Aboriginal persons [19-22]) also provides the backdrop to our study. These groups, who are often understudied or absent altogether from the literature [19,23-25], are likely to report group-specific influences (e.g., cultural values and traditions) that may influence their physical activity involvement [24]. Understanding these influences is critical to inform study designs and interventions to enhance physical activity involvement.

The bulk of the research on physical activity correlates has focused on demographic (e.g., age, gender) and psychological (e.g., behavioural control, self-efficacy) variables [16,22]. Increasingly, researchers are recognizing the importance of environmental factors that may be associated with physical activity involvement [16,26]. For example, aesthetics and convenience of access to destinations have been found to be associated with walking in numerous studies [14]. At the same time, safety has been shown to be associated with walking in women but not in men [14]. At present it is unclear what environmental variables are differently associated with physical activity and walking [14], nor how these associations vary across diverse populations [15,19]. Moreover, we do not know how specific environmental features inherent to rural environments can influence physical activity involvement because the rural environment is largely ignored in the literature [21].

Although research into the link between the environment and physical activity is in its early stage and there is no conclusive evidence that improvements to the environment will enhance physical activity, it is clear that people who have access to safe places in which to walk, play, and be active are more likely to be active [27,28]. Given early evidence showing strong associations between environmental correlates and physical activity involvement and/or walking [16,18,29], and the lack of insight into environmental correlates specific to rural-living Aboriginal people, the purpose of this study was to investigate the relationship between Aboriginal community dwelling adults' perceptions of the environment and their physical activity and walking patterns. For the purposes of this study, environmental perception variables are defined as features of the environment that an individual views as affecting their ability to be physically active.

## Methods

### Study context

Moose Factory is an island community of approximately 2,500 people located near the mouth of the James Bay in Canada. Traditionally, this was a summer meeting place as the Cree people would travel to and from harvesting areas, living off the land year round. The Hudson's Bay Company established a fur trading post there in 1673, exposing the Cree population to the European way of life and forever altering their hunter-gatherer lifestyle [30]. During this time, a majority of the population began the transition from a nomadic way of life to that of permanent community settlers who relied heavily on external sources. It became common for the Cree to spend greater periods of time settled at or near the trading post where they would trade traditional tools and animal hides for European tools and food [31,32]. The signing of Treaty No. 9 in 1905 solidified the relationship between the Cree people of Northern Ontario and the Federal Department of Indian Affairs. In 1907 a 'Native reserve' was established and a residential school was operated on the island [32]. Not only were the language and cultural traditions of the Cree affected, but the dietary habits and activity patterns were also influenced greatly during this period of acculturation and westernisation. Lark Ritchie [32], a local anthropologist describes the transition that began with the introduction of a European way of life:

*We became dependent. Slowly; not violently; not abruptly; we continued to hunt and we trapped; but instead of being travellers, some of us came back to the same place. Rather than families travelling together, they stayed near the posts, and the men went out to hunt and trap, and came back. Our trading had become a commercial venture, and as experienced hunters and trappers, we actually profited by our skills. We had come to know 'capitalism' and 'material possession'. Permanent pots and pans, metal spoons and forks, dishes, extra clothing, a permanent roof. Too much to carry in a canoe. Too much to leave if we travelled. We had become dependent on a relationship designed to take us out of our Native land*...

The traditional lifestyle of earning a living and surviving off the land has thus largely passed. More common now are daily hunting trips or trips lasting a few weeks at time. Qualitative and descriptive observation suggest a high calorie diet and sedentary activity patterns characterize the current lifestyle behaviours of many Moose Factory community members [30].

This significant shift in lifestyle brought with it a new era of health challenges for the James Bay Cree and for Aboriginal communities across Canada. In 1998, the direct age-standardized prevalence of diabetes in Moose Factory was 10.3%, more than double the estimated rate of diabetes in the Canadian population (≈ 5%) [33]. Additionally, the 2002/03 First Nations Regional Longitudinal Health Survey, which gathers information about the health, wellness, and health determinants of First Nations living in First Nations communities across Canada, estimated the rate of diabetes in First Nations communities to be at 14.5% [34].

Trends in activity and diet are also of concern. Although the 2001 Aboriginal People's Survey suggests that 56% of First Nations people in Canada participate in sports, games, or recreational activities [35], other reports suggest that 47% of Aboriginal people in Canada are moderately physically active [36] and that in 2003, only 21.3% of the First Nations population in Canada participated in 'sufficient physical activity' (30+ minutes of activity with increased heart rate and breathing 4+ days a week). This report also revealed that 32.5% of First Nations persons consume soft drinks on a daily basis and 7.9% consume fast food on a daily basis [34].

Moose Factory community members who have become increasingly concerned with the high prevalence of Type 2 diabetes in their community have recently sought out the expertise of an established diabetes prevention team to aid in the development of a diabetes prevention program for their community. The Kahnawake Schools Diabetes Prevention Project Centre for Research and Training in Diabetes Prevention, the research and training arm of the Kahnawake Schools Diabetes Prevention Project (KSDPP [37,38]), is collaborating with the community to address diabetes through initiatives such as this one.

### Recruitment

Two-hundred and seventy-four community members were recruited through a booth set up inside the only shopping complex on the island, posters displayed strategically throughout the community, and advertisements placed on the local television channel and on the two local radio stations. Study eligibility criteria included the following: be 18 years of age or older, have lived in the community for five years or more, and be physically able to participate in walking or regular physical activity. Although we did not specifically ask about ethnicity, we are confident that our sampling strategy yielded a majority of people from Cree descent because most of the transient population (i.e., living in the community for less than five years) would have been excluded from our sample. Eleven people were excluded based on their self-reported inability to partake in physical activity.

### Participants

One hundred and thirty women (mean age = 35.6 years, SD = 12.3) and 133 men (mean age = 36.3 years, SD = 12.7) volunteered to participate in this study. Given that Statistics Canada did not completely enumerate Moose Factory during the 1996 and 2001 Censuses [39], it is not possible to confirm the representativeness of our sample. However, based on numbers compiled by several different community groups (e.g. Moose Cree Band Council, the Weeneebayko General Hospital) we estimate that this sample of 263 adults represents just over 10% of the entire Moose Factory population. Community members were informed that their participation was completely voluntary and anonymity was assured. Verbal informed consent was obtained prior to participation in accordance with the KSDPP Code of Research Ethics [40] and the relevant university research ethics board. Community members who completed the questionnaire were entered into a cash draw for $100.00.

### Measures and Procedures

A brief 15-item survey was developed to assess environmental perceptions, walking, and physical activity. Survey items were drawn from standardized, validated questionnaires [29,41] and refined with community input. For example, in response to community members' request that the survey be brief (in order to reduce participant burden) we selected a reduced number of items, deemed by the group to be most relevant for Moose Factory. Respondents completed the survey in approximately six minutes at a table set up in a quiet area in the shopping complex. This location was chosen following advice from community members who considered our initial data collection protocol, a door-to-door survey, as being too invasive and presumptuous.

#### Demographics and body mass index

Participants' self-reported age, gender, height and weight were recorded. Height and weight were used to calculate body mass index (BMI) [weight (kg)/height (m^2^)].

#### Physical activity

Items from the Godin Leisure-Time Exercise Questionnaire [41] were used to assess physical activity involvement. Questions required participants to separately recall frequency and duration of vigorous, moderate, and light physical activity involvement over the past 7-day period. Two week test-retest reliability coefficients for vigorous, moderate, light intensities have been reported to be: 0.94, 0.46, 0.48 respectively [41]. Two outcome variables were constructed from this assessment: 1) total weekly physical activity involvement (i.e., total number of minutes spent in strenuous, moderate, and light over the previous 7 days), and 2) meeting physical activity recommendations outlined in Canada's Physical Activity Guide to Health Living (i.e., >30 minutes of moderate and/or > 60 minutes of light physical activity five or more days a week [42]).

#### Walking

Self-reported frequency (times per week) and duration of walking (in minutes, at any intensity) in the past 7 days were multiplied to yield two outcome variables: 1) total weekly walking (i.e., total number of minutes spend walking over the previous 7 days), and 2) meeting physical activity recommendations (i.e., engaging in a minimum of 60 minutes of walking at least five days a week [42]).

#### Environmental perceptions

Environmental attribute items were selected from a review of studies investigating environmental factors that have been found to be associated with adults' involvement in physical activity [18,29]. Three separate items were used to assess community features: (a) 'convenience': convenience of shops stores and other places to buy things near by, (b) 'safety': safety of the community for walking, and (c) 'aesthetics': interesting features to look at while walking in the community. Each item was measured on a four-point scale (1 = strongly agree to 4 = strongly disagree). Two items were used to assess home-level environmental supports: (a) owning home exercise equipment, and (b) having sneakers/shoes comfortable enough for walking short distances. Reported absence/presence of these items were recorded.

### Analyses

Descriptive statistics (means, standard deviations, frequencies, percentages) were calculated. Bivariate relationships between demographic variables (i.e., age, gender, and BMI) and meeting walking and physical activity recommendations were examined by conducting chi-square analysis (*p *< .05). Hierarchical multiple regressions were conducted to determine whether demographic and environmental variables were associated with total weekly walking and total weekly physical activity involvement. In each regression, gender was entered in the first step, followed by age and then BMI in the second and third steps, respectively. In the final step, community aesthetics, convenience, and safety, home exercise equipment, and walking/running shoe ownership were entered together.

Given that degree of intensity is lost in the all-intensity measure of total weekly physical activity involvement, we also conducted intensity-specific analyses for each of the following: light weekly minutes, moderate weekly minutes, and strenuous weekly minutes of physical activity. Our rationale for this subset of analyses is founded on evidence suggesting that physical activities of varying intensity may have different predictors and moderators [28]. To examine the effects of perceived environmental variables on physical activity for each different intensity, only those persons reporting involvement in a given intensity were included in the analysis. In other words, those persons reporting zero minutes of strenuous physical activity were excluded from the analysis examining weekly minutes of strenuous physical activity, as we were explicitly interested in the variables affecting those actually partaking in the activity intensity being investigated.

## Results

Eighty (30.4%) of the 263 respondents reported meeting current Canadian physical activity recommendations (i.e., ≥ 30 minutes of moderate physical activity at least five days per week or ≥ 60 minutes of light physical activity at least five days per week) [42]. Similarly, 79 respondents (30%) reported walking enough to meet physical activity recommendations (≥ 30 minutes of walking at least five days per week) while 212 respondents (80.6%) reported walking on at least one occasion throughout the week. Over one third of study participants (i.e., 34.5%) were considered class I obese (i.e., BMI over 30 and less than 35) and an additional 21% were considered class II obese or above (i.e., BMI greater than 35; [43]). Total weekly physical activity involvement decreased with increasing BMI χ^2 ^(4,N = 253) = 11.72, *p *= .02, and total weekly walking decreased with increasing age χ^2 ^(2,N = 263) = 7.18, *p *= .03, and increasing BMI χ^2 ^(4,N = 253) = 19.59, *p *= .001. Men were more likely than women to report sufficient levels of physical activity involvement χ^2 ^(1,N = 263) = 5.14, *p *= .016, but no more likely to report sufficient levels of walking (*p *> .05). See Table 1.

**Table 1:** Participant Characteristics

Variable	N	Active (%)^a^	Sufficient Walking (%)^b^
**GENDER**			
Women	133	24.06	31.58
Men	130	36.92	28.46
			
**AGE**			
18–34	130	36.15	37.69
35–54	110	26.36	22.73
≥ 55	23	17.39	21.74
			
**BMI**			
18.5–24.9	29	48.28	62.07
25–29.9	83	37.35	33.73
30–34.9	87	24.14	24.14
35+	53	18.87	18.87

^a ^Active = ≥ 30 minutes of moderate physical activity at least five days per week or ≥ 60 minutes of light physical activity at least five days per week

^b ^Sufficient walking = ≥ 30 minutes of walking at least five days per week

### Walking

Total weekly walking variable scores were transformed using square root transformation to normalize the positively skewed data. Predictor variables safety and aesthetics were also transformed using square root transformations [44]. Hierarchical multiple regression analysis with the dependent variable square root of total weekly walking revealed significant associations with age, BMI, and perceived environmental variables in the final model (see Table 2). Gender was entered in the first step, but did not contribute significantly to the variance (*p *> .05). In the second step, age was entered and it accounted for 3.3% of the variance F (1,253) = 8.59 (*p *< .05). BMI, entered in the third step, explained an additional 5.9% of the variance in total weekly walking F (1,252) = 16.44 (*p *< .001). In the final step, perceived environmental variables accounted for an additional 5.3% of the variance F (5,247) = 3.04 (*p *< .05). Both square root of safety and aesthetics were significantly related to total weekly walking when all other variables were controlled for (p < .05; Beta 0.130, 0.186 respectively).

**Table 2:** Results of hierarchical multiple regression to explain walking and physical activity by perceived environmental variables

Step	Variable in model	Adjusted R^2 ^change	Standardized Beta
Associations with total minutes of walking per week (n = 262)			
1.	Gender	.001	.084
2.	Age	.037	-.140*
3.	BMI	.079	-.256**
4.	Home equipment		-.004
	Having shoes		.090
	Sqrt Aesthetics	.052	.186*
	Convenience		.048
	Sqrt Safety		-.130*
Associations with total minutes of all intensity physical activity (n = 262)			
1.	Gender	.034	-.163*
2.	Age	.039	-.180**
3.	BMI	.029	-.164*
4.	Home equipment		-.047
	Having shoes		.019
	Sqrt Aesthetics	.017	.187
	Convenience		-.021
	Sqrt Safety		-.094
Associations with total minutes of strenuous physical activity per week (n = 120)			
1.	Gender	.015	-.151
2.	Age	.024	-.187
3.	BMI	.029	-.163
4.	Home equipment		-.224
	Having shoes		.029
	Sqrt Aesthetics		.124
	Convenience		.032
	Sqrt Safety	.072	-.030
Associations with total minutes of moderate physical activity per week (n = 165)			
1.	Gender	.014	-.090
2.	Age	.022	-.127
3.	BMI	.004	-.084
4.	Home equipment		.114
	Having shoes		.049
	Sqrt Aesthetics	.028	.027
	Convenience		.001
	Sqrt Safety		-.125
Associations with total minutes of light physical activity per week (n = 242)			
1.	Gender	.001	.007
2.	Age	.013	-.090
3.	BMI	.017	-.147*
4.	Home equipment		.042
	Having shoes		.017
	Sqrt Aesthetics	.007	-.031
	Convenience		-.002
	Sqrt Safety		-.060

*p < .05, **p < .001; Sqrt= square root transformation

### All intensity physical activity

A second hierarchical multiple regression analysis with the dependent variable square root of all intensity physical activity (weekly total; transformed due to being positively skewed [44]) revealed significant associations with gender, age, and BMI (see Table 2). Gender was entered in the first step and accounted for 3.4% of the variance F (1,251) = 8.74 (*p *< .05). Age was entered in the second step and explained 3.9% of the variance F (1,250) = 10.55 (*p *< .001). BMI, entered in the third step, accounted for 2.9% of the variance F (1,249) = 8.14 (*p *< .05). Perceived environmental variables were entered in the final step, but did not contribute significantly to the variance (*p *> .05).

Strenuous, moderate and light physical activity

The log of weekly strenuous minutes of physical activity was entered into a hierarchical multiple regression as the dependent variable (n = 120). No demographic or perceived environmental variables contributed significantly to the variance (*p *> .05). The log of weekly moderate minutes of physical activity was entered into a hierarchical multiple regression as the dependent variable (n = 165). No demographic or perceived environmental variables significantly contributed to the variance in moderate physical activity (*p *> .05). Using the log of weekly minutes of light physical activity as the dependent variable (n = 242) revealed significant associations with BMI. Gender and age, respectively entered in steps one and two did not significantly contribute to the variance (*p *> .05). BMI, entered in the third step, accounted for 1.7% of the variance F (1,232) = 4.11 (*p *< .05). Perceived environmental variables, entered in the last step, were not significantly related to involvement in light physical activity (p > .05).

## Discussion

The purpose of this study was to investigate the relationship between Aboriginal community dwelling adults' perceptions of the environment and their physical activity and walking patterns. Our examination of participant demographics showed that, consistent with results from the 2002/03 First Nations Regional Longitudinal Health Survey [34], just over half of respondents could be classified as obese [43]. Taking into account that self-reported height and weight are prone to bias, in that persons tend to overestimate their height and underestimate their weight [45], we can assume that these results actually underestimate the prevalence of obesity in our sample. This extreme level of overweight and obesity in our sample merits consideration in light of cross-sectional studies which have consistently shown a graded, negative relationship between physical activity involvement and BMI [16]. The high levels of overweight and obesity observed may be contributing to the low levels of physical activity in this population and must be considered when interventions are designed and implemented.

Consistent with previous findings on walking [46,47], community walking had strong relationships with perceived environmental variables. Specifically, aesthetics and safety were significantly related to walking when all other variables were controlled for. Thus, different aspects of the physical environment appear to influence different types of physical activities, consistent with the notion that environmental factors have their strongest effect on behaviours that occur in those same environments [47]. It makes sense that feeling unsafe (e.g., from wild animals) would have a stronger influence on walking on a community trail than on participating in a yoga class at the local community centre.

The significant amount of variance in behaviour accounted for by perceived environmental variables highlights the importance of considering environmental factors in intervention and determinant studies aimed at increasing the understanding of physical activity involvement. Additionally, this large amount of variance is particularly substantial if it is actually "added" explained variance. In other words, if these perceived environmental features are contributing to the prediction of behaviour above and beyond demographic, psychological, social, and cultural determinants already demonstrated to affect physical activity involvement, [12] this contribution is indeed noteworthy. Furthermore, it is important to consider the implications of these effects once they are multiplied over the entire population [28], where the influence on a population's physical activity level could be substantial [14]. Future work needs to examine integrated intervention models that include intrapersonal, interpersonal, and environmental correlates specific to Aboriginal adults living in rural communities to determine the relative contribution of each ecological dimension to people's involvement in walking and physical activity.

Our findings add to and are consistent with previous research showing that environmental aesthetics are related to increased walking in the community [25,46-50]. Given that many rural communities such as Moose Factory are characterized by pleasing natural environmental features (e.g., waterfront vistas), this may represent a promising avenue for encouraging physical activity in specific community locations and/or for improving the aesthetic appeal of other community locations.

Sharpe and colleagues also found that persons perceiving greater safety were more likely to meet physical activity recommendations [48]. Unlike the present study, however, the authors did not differentiate between walking and total physical activity. More research is needed to investigate the association between different types of physical activity, including walking, with perceived safety before conclusions can be drawn. While safety does appear to be a correlate of physical activity across a wide range of environments (e.g. rural and urban) [51], we can speculate that its importance in a northern-rural environment is distinctive from that of most urban environments. For example, while it is highly unlikely that an individual in an urban centre refrains from physical activity to avoid environmental threats such as bears, this threat is real for people living in communities such as Moose Factory. It therefore follows that environment-specific strategies will need to be developed to address safety issues inherent to a given environment. It is clear that additional qualitative research is required to further investigate specific safety issues (e.g. from violent crime, bears, dogs, etc) relevant to physical activity in different types of Aboriginal communities.

That perceptions of the surrounding environment are more influential to walking than to general levels of physical activity is fairly consistent with the literature and supports the notion that environmental factors will have their strongest effects on behaviours that occur in those same environments [16,23,51]. This confirms that the unique environment of a remote rural community needs to be accounted for in intervention plans to promote walking. Intervention strategies might include ways to counter unsafe situations (e.g., walking with a group rather than alone) or ways in which to enhance safety (e.g., fencing an outdoor area where walking can take place).

To better understand the factors that influence community members' physical activity and walking, a wider range of correlates must be investigated (e.g. traditional beliefs). An increased understanding of how these correlates interact to influence behaviour in this understudied segment of the population is needed. Research conducted by Lavallée suggests that including Aboriginal core values, beliefs and healing practices in physical activity programming will increase Aboriginal people's adherence and connection to such programs [52]. Including cultural values such as smudging, tobacco bundles, and purification ceremonies in physical activity programs will encourage a more wholistic (this spelling is used to reflect the Aboriginal philosophy of the "whole" in which "everything is related by virtue of shared origins and in which, by extension, the human being is considered an entire whole, that is, mentally, physically, spiritually, and emotionally..." [53]) view of physical activity and allow healing at multiple levels (e.g. physical, mental, emotional, and spiritual) [52].

Our results suggest that one's environment is an important factor to consider when physical activity participation is in question. Environment factors should not be ignored, but rather be considered in addition to and not instead of other social and individual level variables. Simply creating supportive environments for physical activity may not be sufficient and intervention studies need to consider individual, social environmental and environmental variables [54].

Some limitations to the current study deserve mention. The cross-sectional design of the investigation limits the conclusions that can be drawn and the use of convenience sampling limits the generalizability of results. Objective measures of the environment were not collected, as the study was designed to investigate community members' perceptions of their environment. Subsequent studies should investigate the relationship between perceived and objective measures of the environment. Reliance on self-report to assess physical activity and walking may have yielded inflated estimates of these behaviours. Similarly, self-reported height and weight may have led to an underestimation of levels of overweight and obesity. Despite these limitations, our findings provide new information concerning an environment and population segment that has been largely understudied. Additionally, the results provide supportive evidence in an area where there is growing interest, but limited published data [45]. The information gathered in this study can be utilized by community members to design more appropriate and effective physical activity interventions.

Results indicate that the relationship between physical activity and the environment is complex whereby different environmental variables appear to be associated with different types of physical activities. Further investigation is needed into methods for altering persons' perception of their community environment. Possibly improving community members' perceptions concerning safety and aesthetics could foster increased physical activity involvement [55].

### Recommendations for community intervention

A compilation of the information generated by this investigation provides valuable direction for the newly formed diabetes prevention team in this community and anyone else planning interventions in this community or in other rural Aboriginal communities in Canada. Our results suggesting that perceived environmental aesthetics and safety are both related to walking behaviour in the community might serve as a starting point for intervention design. A summary of the information garnered was reported back to the community through a community newsletter, a presentation to the local health committee and diabetes prevention team and through posters displayed throughout the community. In addition, community member's input and approval was solicited throughout the writing process.

## Conclusion

These findings indicate that walking appears to be influenced differently by the surrounding environment than by general physical activity involvement. As well, similar determinants appear to affect individuals living in very different environments, but the underlying reasons may be quite varied and further investigation is warranted. Study results provide much needed information concerning the determinants that mediate physical activity participation in a rural environment with an understudied population segment. Several significant associations were documented, demonstrating the importance of considering environmental factors in physical activity intervention and correlate studies.

Because current findings suggest a possible variation in correlates across age, gender, and BMI, specific consideration for each of these attributes is recommended in subsequent investigations. Furthermore, the findings underscore the necessity of additional research to further the understanding of how perceived convenience; aesthetics, safety, exercise equipment in the home, presence or absence of comfortable shoes, and other environmental factors may influence physical activity levels in different population strata across varied environments. Future investigations should include a wide range of possible correlates in prospective and intervention designs that are behaviour-specific. Attributes specific to a particular physical activity type (e.g. intensity level) in a particular context and setting also need to be considered.

## Competing interests

The author(s) declare that they have no competing interests.

## Authors' contributions

AK participated in the conception and design of the study, the acquisition of data, analysis and interpretation of data, and the drafting of the manuscript. LL was involved in the conception and design of the study, analysis and interpretation of data, and the drafting of the manuscript. VW participated in the design of the study and the acquisition of data. JRW was involved in the analysis and interpretation of data, performed the statistical analysis, and critically revised the manuscript. All authors read and approved the final manuscript.
